# Socially cued developmental plasticity in web-building spiders

**DOI:** 10.1186/s12862-016-0736-7

**Published:** 2016-08-26

**Authors:** Rainer Neumann, Jutta M. Schneider

**Affiliations:** Zoologisches Institut, Universität Hamburg, Martin-Luther-King-Platz 3, 20146 Hamburg, Germany

**Keywords:** Adaptive plasticity, Environmental uncertainty, Density cues, Information use, Male-male competition, Nephilidae, SSD

## Abstract

**Background:**

Socially cued anticipatory plasticity (SCAP) has been proposed as a widespread mechanism of adaptive life-history shifts in semelparous species with extreme male mating investment. Such mating systems evolved several times independently in spiders and male reproductive success should critically depend on timely maturation and rapid location of a receptive and, ideally, virgin female. We experimentally investigated socially cued anticipatory plasticity in two sympatric, closely related *Nephila* species that share many components of their mating systems, but differ in the degree to which male reproductive success depends on mating with virgin females. Juveniles of both species were reared either in the presence or absence of virgin female silk cues. We predicted strong selection on socially cued plasticity in *N. fenestrata* in which males follow a highly specialized terminal investment strategy, but expected a weaker plastic response in *N. senegalensis* in which males lost the ability to monopolize females.

**Results:**

Contrary to our predictions, *N. fenestrata* males presented with virgin female silk cues did not mature earlier than siblings reared isolated from such cues. Males in *N. senegalensis*, however, showed a significant response to female cues and matured several days earlier than control males. Plastic adjustment of maturation had no effect on male size.

**Conclusions:**

Our results indicate that a strong benefit of mating with virgins due to first male sperm priority does not necessarily promote socially cued anticipatory plasticity. We emphasize the bidirectional mode of developmental responses and suggest that this form of plasticity may not only yield benefits through accelerated maturation, but also by avoiding costs of precipitate maturation in the absence of female cues.

## Background

In most organisms, genetically identical individuals develop markedly different phenotypes when exposed to different environments [1–6] and such plastic modifications of morphology, physiology, life-history or behavior have been frequently shown to be adaptive, yielding increased fitness returns under specific conditions [7–9]. Juvenile development, maturation, and the period of reproduction in many animal species follow recurrent seasonal gradients [10, 11], thus it is crucial to adjust one’s own reproductive period to the opposite sex, particularly in semelparous species experiencing only a single reproductive episode. Often thermal threshold values [12] or photoperiod changes are used as indicators of large-scale seasonal progression [13]. However, fluctuations of external conditions may alter the density and structure of a population [14–16] and sex-specific differences in developmental rates or mortality will further add temporal demographic variation. Such local differences are difficult to predict from large scale cues and plastic adjustment of life-history traits based on local information might be advantageous [17].

Recent studies have highlighted the role of social cues in adaptive life-history shifts, for example, in response to the density of conspecifics [18, 19]. Since accelerated or delayed juvenile development in response to conspecific cues precedes its fitness-relevant effect at the stage of maturity, these mechanisms have been termed ‘socially cued anticipatory plasticity’ (SCAP; [20]). Socially cued developmental tactics are hypothesized to be more common than currently appreciated [20], but plasticity can also involve fitness costs [21, 22] that may constrain the evolution of such traits. Moreover, an established cue from the social environment that benefits plastically responding individuals in a single species may not be similarly relevant in related species, as the value of particular cues will strongly relate to specific features of the mating system under study [20].

Many spiders show extreme reversed sexual size dimorphism (SSD; [23, 24]) and specialized male strategies to maximize and protect paternity, such as self-sacrifice [25–27], genital plugging [28–30] or remote copulation [31]. In such species, males generally benefit most from locating a virgin female [32–34], which may impose selection on males to mature earlier. The transition to the reproductive stage, however, is critical because maturing males lose the ability to capture prey on their own. Since adult males are restricted to feeding opportunistically on the females’ prey [35, 36], precipitate maturation may also be unfavorable.

Spider males perceive conspecific females using one or more sensory modalities, but most species lack acute vision and have to rely on mechanical or chemical signals that indicate the presence of receptive females [37, 38]. Female sex pheromones, both volatile and incorporated in female silk, have been shown to serve this function in orb-web spiders [39–41], but only one study on the Australian red-back spider *Latrodectus hasselti* provides evidence that female pheromones induce adaptive developmental plasticity in males [42]. Like most animals [43], males in this species have to trade-off developmental time against growth, as fast-maturing males stay relatively small, but intense contest competition shifts fitness payoffs to larger males [44].

Specialized male mating strategies that allow maximizing paternity with a single or very few females and constitute a very high mating effort have evolved at least four times independently in different spider families [45, 46]; providing ideal model systems to investigate whether such characteristics generally promote socially cued anticipatory plasticity. Differences in specific mating traits may affect selection on such mechanisms and a comparative approach may help to relate the magnitude of plastic responses to the associated adaptive value.

The golden-silk spider genus *Nephila* is an established model system in sexual selection research, which has been used to study, for example, female-biased SSD [24], sexually selected life-history traits [47, 48], male-male competition [49–52], and intrasexual size variation [53, 54]. Especially male size varies greatly in some species [55, 56]. Socially cued developmental plasticity may increase male size variation; even more so as the capacity for plastic modifications may differ between genotypes [9]. *Nephila* females build large orb-webs, whereas males cease web-building after reaching maturity to search for females [57]. Population densities change in the course of the season [58–60], but in addition, local environmental conditions cause strong between-year variation in some species [61]. Socially cued plasticity could serve to adjust male development to female availability and male-male competition, but experimental work is required to determine whether predefined cues induce the expected developmental modifications [20].

We examined the capacity of males to optimize the timing of maturation in response to female silk cues using two sympatric *Nephila* species that are exposed to almost identical abiotic cues of seasonal changes in their natural habitat, *N. fenestrata* and *N. senegalensis*. Both species are generally similar in their reproductive biology, but differ in certain aspects of male mating strategies. *N. fenestrata* males follow a terminal investment strategy aimed at monopolizing a single female by means of mate plugging through copulatory organ breakage [28], whereas in *N. senegalensis*, males do not produce mating plugs and each male is able to fertilize up to four females [62]. Males in this species adopt flexible mating tactics including male mate choice and polygyny, which reduce the imbalance in reproductive success between males that encounter a virgin or a non-virgin female first [55]. Hence, although males in both species prefer mating with virgins [55, 63, 64], life-time fitness in *N. fenestrata* more strongly depends on locating an unmated female. These differences affect the value of prospective mates and are expected to generate dissimilar selection on socially cued anticipatory plasticity; an assumption in line with a field study on two other orb-web spider species suggesting anticipatory plastic responses to female densities in the monogynous *N. plumipes*, but not in *Argiope keyserlingi*, in which males are usually bigynous [17].

We reared juvenile *N. fenestrata* and *N. senegalensis* under standardized conditions in climate-control chambers, presenting spiders in the experimental treatment with virgin female silk. We expected the highly specialized, terminally investing *N. fenestrata* males to accelerate their development in the presence of virgin female silk cues. Males were supposed to mature earlier, but at smaller size, than siblings in the control treatment without virgin female cues. In *N. senegalensis*, males are often polygynous and depend less on locating a virgin female, hence we predicted a weaker developmental response in this species.

## Methods

### Study animals

Spiders used in this study were F2 offspring descending from females that were collected at Mawana Game Reserve, Zululand District, KwaZulu-Natal, South Africa in 2012 (permit OP 990/2012 from EZEMVELO KZN WILDLIFE PERMITS OFFICE). All families of study animals were derived from mating virgin individuals from different maternal lines. Six family lineages were used in each species comprising 23.7 ± 5.6 individuals per family in *N. senegalensis* and 25.5 ± 5.4 individuals per family in *N. fenestrata*. We reared hatchlings communally at first, and separated them after approximately two additional molts to maintain them in 200 ml plastic cups, which were turned upside down. Spiders were kept under standardized conditions in our main laboratory [63] before being transferred to the experimental rooms. The experiments took place at the Zoological Institute, University of Hamburg, between May 23 and August 25, 2013.

### Experimental setup and treatments

Spiders were kept in two climate-control chambers, measuring approximately 1.9 m × 4.3 m × 2.4 m each, in order to control temperature, relative humidity, light-dark cycle, and light intensity. Climate-control chambers (Weiss Umwelttechnik GmbH, model type WK 21’/5–40) featured identical technical specifications. Both devices were contemporaneously installed, calibrated and put into operation by the manufacturer’s expert staff in 2012.

For each of our study species, we established an experimental treatment in which adult virgin females’ silk was introduced to the spiders’ rearing cups (referred to as the Female cues treatment). Thereby, we presented the study animals with potential contact pheromones or any properties of silk that may indicate the presence of adult females. In a control treatment, spiders were reared isolated from adult virgin female silk cues (referred to as the No cues treatment). To exclude long distance perception of female silk cues in the control treatment, we arranged the Female cues treatments for both of our study species simultaneously in one climate chamber and used the second chamber for both No cues treatments. Each climate chamber was equipped with six bottom shelves and six top shelves. A tubular fluorescent daylight lamp was mounted above each shelf with a distance of 60 cm. We placed up to twenty-seven rearing cups on each shelf with an equal distance of approximately 15 cm between cups. Prior to the transfer of study animals to the experimental treatments, we adjusted climate-control chambers to provide identical conditions of temperature, relative humidity, and light regime. Temperature and humidity were regulated corresponding to periods of artificial daytime and night-time throughout the experiment; i.e., temperature was set to 26 °C during lighting periods and 21 °C during dark periods. We set daytime humidity to 50 % and night-time humidity to 70 %, respectively. These conditions fit well within the range in both species’ habitats. In the beginning of the experiment, we used a 14:12 h light-dark cycle and reduced the daily lighting duration by 10 min each week to simulate a decrease in day length, which both of our study species experience during summer and autumn in their habitats of origin.

### Transfer of study animals to climate-control chambers

*Nephila fenestrata* study animals were transferred to the climate chambers on May 23; *N. senegalensis* were transferred on May 26/27. We used a split brood design and allocated equal numbers of randomly chosen individuals from each family lineage to each treatment. After the transfer had been completed, we checked all study animals for presence and condition on the following day and replaced a small number of spiders that had died or vanished from the rearing cups. No study animals were replaced at a later date.

### Maintenance and monitoring schedule

The regular monitoring of study animals began on May 29 (defined as the start of the experiment) with the following numbers of study animals: *N. fenestrata*: Female cues treatment: *n* = 156; No cues treatment: *n* = 157; *N. senegalensis*: Female cues treatment: *n* = 162; No cues treatment: *n* = 162. Spiders were fed *Drosophila* flies twice a week on a regular schedule. In the initial stage of the experiment when the spiders were still very small, we used flies that had been killed at −80 °C. When all spiders had reached a minimum body length of approximately 5 mm, we supplemented the diet with live insects. This food supply allowed the spiders *ad libitum* feeding. Water was offered on 6 days per week. At this stage, we checked the animals’ condition four times a week and recorded any cases of death as well as spiders that had vanished from their rearing cups (missing spiders likely dropped from rearing cups during feeding or cleaning of shelves).

### Introduction of female silk cues

As a consequence of female-biased SSD, *Nephila* females take longer to mature than males, so that early maturing males become adults in populations devoid of adult females (protandry; [57, 60]). As our goal in this study was to simulate the beginning of the mating season, we presented males with adult virgin female cues not from the start, but after a period of development in the absence of such cues. In the Female cues treatment, we introduced the first set of silk cues to the rearing cups on days 22/23 from the start of the experiment for *N. fenestrata* and on days 22–24 for *N. senegalensis* (all subsequent sets of silk cues were introduced within one day). We used plastic expansion bolts to present silk samples to the study animals. For this purpose, the expansions of each piece were spread, resulting in a Y-shaped object, which we put up in vertical position using a base of potting clay. These silk fixtures measured 5.5 cm in height. For acquiring silk cues, we used female webs the spiders had built into 40 cm × 40 cm-sized Perspex frames. Webs had usually been newly built in the previous night, but were at most two days old. Females were removed from their webs and the frames were taken to the female cues chamber. We then twisted a few silk threads from the web’s moistened catching spiral around the upper expansions of each silk fixture and used fine scissors to dissect the threads from the web. One silk fixture was placed under each rearing cup, so that the spider inside could easily access the silk threads, especially with its pedipalps and forelegs, bearing the most important sensory organs to perceive physical and chemical cues [65, 66]. Fresh silk cues were introduced on a weekly schedule (on days 29, 36, 43, 50, 57, 64, 71, 78, and 85 from the start of the experiment). On the previous day, we removed all silk fixtures from the rearing cups and cleaned the shelves in the experimental rooms. Each object was cleaned of silk with alcohol and air-dried prior to reuse. In order to standardize experimental conditions, we placed identical objects free of silk under the rearing cups in the No cues treatments. Silk cues were acquired from twenty-four adult virgin female *N. fenestrata* (up to four per turn) and thirty-three *N. senegalensis* (up to six per turn). Females originated from eleven family lineages in *N. fenestrata* and twelve family lineages in N. senegalensis. Average female adult age (days passed from date of maturity) at the time of web production was 13 days (range: 2–30 days) in *N. fenestrata* and 11.5 days (range: 2–29 days) in *N. senegalensis*. How many times a male received fresh silk cues depended on individual developmental durations. Those males in the Female cues treatments that were used in our analysis received fresh cues 5.4 ± 0.1 times in *N. fenestrata* and 6.4 ± 0.1 times in *N. senegalensis* (range in both species: 3-8 times). Individual silk cues were obtained from a female unrelated to the cues-receiving male (48 % of cues in *N. fenestrata* and 45 % of cues in *N. senegalensis*) or from a female that had one parental lineage in common with the cues-receiving male (52 % of cues in *N. fenestrata* and 53 % of cues in *N. senegalensis*). In < 1 % of cues in *N. fenestrata* and 2 % of cues in *N. senegalensis*, we could not avoid using silk from females that had both parental lineages in common with the cues-receiving male. No male received cues from related females only. With the first implementation of female silk cues, we adjusted the monitoring of study animals and checked the individual state of development on six days per week. For each male, we recorded the duration of development from the start of the experiment to maturity and the duration of the subadult instar (i.e., the last developmental stage; subadult males can easily be detected by the swollen palp tarsi indicating the ongoing transformation into copulatory organs). Juvenile females were immediately removed from the study when they were clearly discernible (body length ≥ approximately 12 mm, pedipalps unmodified).

### Statistical analyses

We defined the start of the experiment as the first monitoring of study animals after being transferred to the climate-control chambers (May 29). In *N. fenestrata*, some males matured before the first introduction of female silk cues had been completed (June 21). These males were excluded from the analyses (predefined female cues chamber: *n* = 5; no cues chamber: *n* = 9). In each of our study species, we analyzed effects of our experimental treatment (Female cues/No female cues) on male development with separate linear mixed models performed in R 3.2.4 (R Development Core Team 2016). Dependent variables were (1) Duration of development from the start of the experiment, (2) Duration of subadult stage, (3) Adult size, and (4) Adult weight. The study animals’ family lineage was included as a random effect. We tested for statistical significance of Treatment using ANOVA model comparisons with *χ*^2^ tests between the full model and a model that had the variable removed. Using the same dependent variables, we conducted generalized linear models in JMP IN 7.0 (SAS Institute Inc., Carey, NC, USA) to test for an interaction between Treatment and Family lineage. Models were fitted with normal error structure and identity-link function. We removed the interaction term if it was non-significant (α = 0.05) while retaining both main effects in the final models. Developmental durations were log-transformed to improve model fit. Descriptive statistics are given as mean ± standard error. Within experiments, sample sizes may differ due to missing data.

## Results

We performed linear mixed models to test effects of our experimental treatment on male development and growth. The models clearly revealed a significant influence of our treatment on the duration of development in *N. senegalensis*. Males in the Female cues treatment matured two to five days earlier, on average, than males in the No cues treatment and the mean duration of the subadult stage alone differed by one and a half to two days (ANOVA model comparisons: Duration of development from the start of the experiment: *χ*^2^ = 10.563, *p* = 0.001; Duration of subadult stage: *χ*^2^ = 29.724, *p* < 0.001; Table 1). However, shortened development did not translate into different male size or body mass (ANOVA model comparisons: Adult size: *χ*^2^ = 1.134, *p* = 0.287; Adult weight: *χ*^2^ = 2.586, *p* = 0.108; Table 1). Contrary to our predictions, in *N. fenestrata*, there were no significant differences in various life-history parameters between males presented with virgin female silk cues and those reared in the absence of such cues (ANOVA model comparisons: Duration of development from the start of the experiment: *χ*^2^ = 0.006, *p* = 0.939; Duration of subadult stage: *χ*^2^ = 1.632, *p* = 0.202, Adult size: *χ*^2^ = 0.528, *p* = 0.467; Adult weight: *χ*^2^ = 1.629, *p* = 0.202; Table 1).

**Table 1:** Developmental parameters of male *Nephila fenestrata* and *N. senegalensis* reared in different experimental treatments

	*N. fenestrata*			*N. senegalensis*		
	No female cues	Female cues	*n*	No female cues	Female cues	*n*
Duration of development (start to maturity) [d]	57.36 ± 0.78	57.18 ± 0.86	153	68.83 ± 0.86	65.36 ± 0.63	142
Duration of subadult stage [d]	18.51 ± 0.18	18.26 ± 0.15	153	21.12 ± 0.25	19.38 ± 0.21	142
Adult size/patella-tibia [mm]	5.69 ± 0.09	5.65 ± 0.09	150	4.74 ± 0.09	4.62 ± 0.1	132
Adult weight [mg]	18 ± 0.58	18.22 ± 0.56	153	22.46 ± 0.66	21.06 ± 0.7	141

**Fig. 1:**
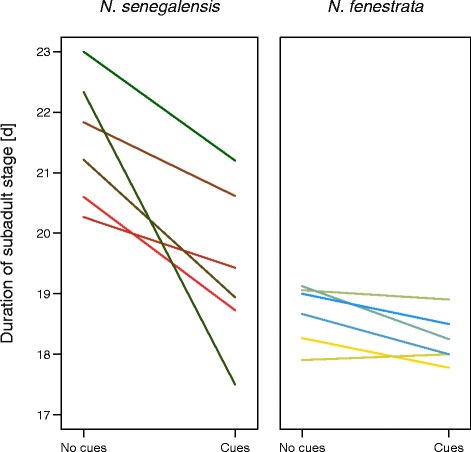
Duration of the subadult instar (i.e., the last developmental stage preceding maturity) in the presence or absence of virgin female silk cues compared between male *Nephila senegalensis* and *N. fenestrata*. Graphs illustrate mean developmental durations according to family lineages

We ran additional generalized linear models to analyze potential family-specific variation of developmental plasticity. In *N. senegalensis*, the response toward a shortened development was present in all family lineages (Fig. 1). The interaction between Family lineage and Treatment, however, was always found to be non-significant at the 5 % level; although developmental responses varied considerably between families (Fig. 1, Table 2). Corroborating mixed model results, the GLMs showed that in *N. fenestrata*, only Family lineage predicted developmental durations, size, and weight, while Treatment had no effect (Table 2). In contrast, both Family lineage and Treatment significantly determined developmental durations in *N. senegalensis* (Table 2).

**Table 2:** Effects of family lineage and treatment on developmental parameters in *Nephila fenestrata* and *N. senegalensis*

Explanatory variable	Duration of development (start to maturity)			Duration of subadult stage			Adult size			Adult weight		
	*χ* ^2^	*p*	*df*	*χ* ^2^	*p*	*df*	*χ* ^2^	*p*	*df*	*χ* ^2^	*p*	*df*
*N. fenestrata*												
Family lineage	50.65	**<.0001**	5	14.6	**0.012**	5	67.07	**<.0001**	5	61.86	**<.0001**	5
Treatment	0.002	0.961	1	2.08	0.15	1	0.73	0.391	1	2	0.158	1
Family lineage *Treatment	2.101	0.835	5	2.332	0.807	5	1.588	0.903	5	1.704	0.888	5
*N. senegalensis*												
Family lineage	21.89	**0.0006**	5	22.23	**0.0005**	5	37.56	**<.0001**	5	40.77	**<.0001**	5
Treatment	11.34	**0.0008**	1	31.01	**<.0001**	1	1.16	0.281	1	2.621	0.105	1
Family lineage *Treatment	10.126	0.072	5	7.924	0.161	5	10.58	0.06	5	9.757	0.082	5

Likelihood-ratio tests and corresponding *p*-values derive from generalized linear models performed in JMP IN 7.0 (SAS Institute Inc., Carey, NC, USA). Non-significant interaction terms were removed from the final models. Developmental durations were log-transformed. Significant *p*-values are shown in bold

## Discussion

Males in one of our study species, *Nephila senegalensis*, plastically adjusted development and matured significantly earlier in response to female silk cues than those reared isolated from such cues. However, we found no developmental response in *N. fenestrata*. While the plastic adjustment of maturation in *N. senegalensis* is in accordance with our predictions, we expected an even more distinct modification of development in the monogynous *N. fenestrata* males whose fitness strongly depend on locating a virgin female [28]. The absence of a plastic response in this species indicates that socially cued anticipatory plasticity is not a universal feature in species with strong developmental differences between the sexes and a high male mating effort.

The plastic adjustment of maturation in *N. senegalensis* is best described as flexibility in the duration of the subadult instar and did not affect male adult size or mass. Males in this species are able to fertilize multiple females [62, 63] and differential mating investment has been identified as an integral part of a flexible mating strategy in this species [55]. In nature, individual males visit up to four females (Neumann & Schneider, unpublished observations); hence adjusted maturation in response to the perception of female cues may increase a male’s chance to locate a virgin female first, and to mate with further females in a period of low or moderate competitive conditions.

Animals in general have to trade-off developmental duration against growth [43]. Increased food intake and delayed maturation will usually result in larger adult body size, which often is an important determinant of male reproductive success in mating systems involving contest competition [67]. This relationship also exists in some web-building spiders [44] and we expected males perceiving the presence of virgin females to mature at smaller size as a consequence of accelerated development. In contrast to a previous study on Australian red-back spiders [42], however, adjustment of maturation in *N. senegalensis* was not achieved by substantially abbreviating development. Rather, the timing of maturation was modified by differences in the duration of the subadult instar and there was no trade-off between adjustment of development and adult body size. Hence, we found no support for socially cued plasticity to contribute to the extreme male size variation observed in many *Nephila* species [47–49, 55]. While males adjusted the timing of maturation in the same direction across family lineages, we also observed considerable variation between lineages regarding the magnitude of plastic responses. Genotype-specific degrees of plasticity in response to an environmental trigger could contribute to phenotypic variation, but our study found little evidence for such interrelations in our model species.

It is important to realize the bidirectional mode of a plastic response; hence not only the expression of a specific modification appropriate to requirements should be beneficial, but also the non-expression of the same modification in the absence of the corresponding trigger. What is to be gained from staying subadult for a male *N. senegalensis* in the absence of adult females?

With sexual maturation, web-spider males undergo drastic changes in terms of morphology, physiology and life-style, solely targeted on reproduction [35]. Adult male spiders lose weight during mate search [68] but are no longer able to build capture webs [51, 57]. In order to maintain a sound physical condition, they depend on stealing prey from female webs [48, 57, 69]. Males maturing without the perspective of locating a female in a short time risk declining physical strength, whereas subadult males residing in their own webs stay relatively safe from predation and may continue feeding on self-captured prey.

Another potential benefit of a delayed maturation may relate to sperm-limitation, which is a universal trait in nephilid spider males [70]. Male *N. senegalensis* produce their lifetime sperm supply in their subadult instar and spermatogenesis is terminated prior to adulthood [62]. Total sperm numbers vary considerably among males [68] and a prolonged subadult instar may allow males to increase sperm quantity to prevail in sperm-competition. Taken together, these arguments support the assumption that *N. senegalensis* males significantly benefit from shifting maturation until mating is about to take place, and not to mature when the probability of finding a female is low.

However, most of these arguments apply to *N. fenestrata* as well and the absence of a plastic response to virgin female cues in this species is puzzling. Owing to mate plugging and copulatory organ breakage, male mating tactics are less flexible than in *N. senegalensis*, and male reproductive success critically depends on the ability of monopolizing a single female [28, 32]. To explain our findings, we might consider between-species differences regarding the value of developmental responses from both male and female perspective. Such differences might be linked to our study species’ ecology, as habitat requirements differ slightly between both species, which could affect the predictability of receptive females. Habitats in *N. senegalensis* range from humid areas to bush savannahs and habitat heterogeneity is reflected in varying population densities (Neumann & Schneider, unpublished observations). Predicting female presence is therefore challenging and selection may favor male ability to fine-tune maturation on a local scale. In contrast, *N. fenestrata* occurs in forested areas (sub *N. pilipes*; [71]) providing relatively constant temperature and humidity, and females typically form dense aggregations in preferred sites (Penney, unpublished observations). Given the rather narrow range of tolerated conditions, female presence may directly be indicated by abiotic large-scale cues and habitat quality, making socially cued anticipatory plasticity less needed in *N. fenestrata*.

Finally, the presence and absence of socially cued plastic responses in the respective species could be explained from the female perspective. We cannot unambiguously relate the developmental response in male *N. senegalensis* to silk-borne pheromones, as the physical properties of adult females’ silk alone could indicate their presence, but females in various web-building spiders use specific chemical signals to attract males and secure a timely mating [72–74]. *N. senegalensis* females are polyandrous [62] and may use pheromone signals to repeatedly attract males. In *N. fenestrata*, however, there may be little need for females to advertise their presence, as males may easily locate them in their spatially-limited habitats; and also because female mating rates are much lower compared to *N. senegalensis*. Pheromone production itself may be costly [75–77] and attracting unwanted males could even decrease female fitness, if there are no significant benefits to be gained from multiple matings [78–80]. Additional research should investigate whether female *N. fenestrata* produce sex pheromones strategically; e.g., only under a high risk of remaining unmated [81].

## Conclusions

Our results suggest that a strong benefit of mating with virgins due to first male sperm priority does not necessarily promote socially cued anticipatory plasticity. Benefits and costs of using and providing information may differ between the sexes. Even if males, in principle, would benefit from plastic life-history shifts, they may sensorially rely on information provided by females. In such cases, the evolution of plasticity may depend on whether females benefit from providing cues, and future studies should take the female perspective into account. In addition, we suggest that the adaptive value of socially cued anticipatory plasticity might not be limited to males that adaptively accelerate development to mature in time, but males that delay maturation in the absence of female cues might also benefit by avoiding potential costs of precipitate maturation.
